# Improving immunogenicity of HIV-1 envelope gp120 by glycan removal and immune complex formation

**DOI:** 10.1186/1742-4690-9-S2-P292

**Published:** 2012-09-13

**Authors:** R Kumar, M Tuen, H Li, DB Tse, CE Hioe

**Affiliations:** 1 New York University School of Medicine, New York, NY, USA; 2 NYU School of Medicine, Department of Medicine, NY, NY, USA

## Background

HIV-1 envelope (Env) gp120 is an important target for neutralizing antibody (Ab) responses against the virus. However, developing gp120 vaccines that elicit potent and broad neutralizing Abs has proven to be a formidable challenge. The envelope gp120 is highly glycosylated and carbohydrate moieties play an important role in modulation of immunobiological property of HIV-1 Env gp120. Previously, removal of a specific N-linked glycan at residue 448 by N to Q mutation (N448Q) has been found to enhance in vitro antigenicity of neutralizing epitopes in the V3 loop. In the present study we examined immunogenicity of mutant gp120 in mice.

## Methods

Two immunization protocols were tested. First, using plasmid DNA expressing gp120BH10 followed by protein boost with QS-21 adjuvant. Second, gp120 was administered as an immune complex with antibody in DDA/MPL adjuvant. Cellular responses were measured using spleen cell proliferation and cytokine production by Bio-Plex multiplex assay. Sera were evaluated for antibody binding via ELISA and neutralization activity via TZM-bl assay.

## Results

With the DNA prime/protein boost protocol, N448Q gp120 mutant induced higher levels of gp120 specific lymphoproliferation and cytokine production as compared to wild type. However, both mutant and wild type gp120s failed to generate anti-V3 Abs and virus-neutralizing Ab response. In contrast, immunization with mutant gp120 in complex with mAb 654 elicited higher titers of neutralizing Abs activity than the wild type counterpart. Neutralizing activity was directed to V3 and other undefined neutralizing epitopes. Improved immunogenicity of immune complexes correlated with increased reactivity and proteolytic resistance of V3 and other Ab epitopes.

## Conclusion

These data demonstrate the advantage of combining site-specific N-glycan removal and immune complex formation as a novel vaccine strategy to improve immunogenicity of Ab epitopes on critical regions of HIV-1 gp120. Importantly, epitope immunogenicity is governed not only by its antigenicity but also by its stability against proteolytic degradation.

